# ANN-Based Estimation of Low-Latitude Monthly Ocean Latent Heat Flux by Ensemble Satellite and Reanalysis Products

**DOI:** 10.3390/s20174773

**Published:** 2020-08-24

**Authors:** Xiaowei Chen, Yunjun Yao, Yufu Li, Yuhu Zhang, Kun Jia, Xiaotong Zhang, Ke Shang, Junming Yang, Xiangyi Bei, Xiaozheng Guo

**Affiliations:** 1 State Key Laboratory of Remote Sensing Science, Faculty of Geographical Science, Beijing Normal University, Beijing 100875, China; chen_xiaowei@mail.bnu.edu.cn (X.C.); jiakun@bnu.edu.cn (K.J.); xtngzhang@bnu.edu.cn (X.Z.); shangke@mail.bnu.edu.cn (K.S.); julming@mail.bnu.edu.cn (J.Y.); xiangyibei@mail.bnu.edu.cn (X.B.); boyxiaozheng@mail.bnu.edu.cn (X.G.); 2 Jincheng Meteorological Administration, Jincheng 048026, China; qxtlyf@163.com; 3 College of Resource Environment and Tourism, Capital Normal University, Beijing 100048, China; yuhu.zhang@cnu.edu.cn

**Keywords:** latent heat flux (*LHF*), artificial neutral network, machine learning methods, triangle cornered hat

## Abstract

Ocean latent heat flux (*LHF*) is an essential variable for air–sea interactions, which establishes the link between energy balance, water and carbon cycle. The low-latitude ocean is the main heat source of the global ocean and has a great influence on global climate change and energy transmission. Thus, an accuracy estimation of high-resolution ocean *LHF* over low-latitude area is vital to the understanding of energy and water cycle, and it remains a challenge. To reduce the uncertainties of individual *LHF* products over low-latitude areas, four machine learning (*ML*) methods (Artificial Neutral Network (*ANN*), Random forest (*RF*), Bayesian Ridge regression and Random Sample Consensus (*RANSAC*) regression) were applied to estimate low-latitude monthly ocean *LHF* by using two satellite products (*JOFURO*-3 and *GSSTF*-3) and two reanalysis products (*MERRA*-2 and *ERA-I*). We validated the estimated ocean *LHF* using 115 widely distributed buoy sites from three buoy site arrays (*TAO*, *PIRATA* and *RAMA*). The validation results demonstrate that the performance of *LHF* estimations derived from the *ML* methods (including *ANN*, *RF*, *BR* and *RANSAC*) were significantly better than individual *LHF* products, indicated by *R*^2^ increasing by 3.7–46.4%. Among them, the *LHF* estimation using the *ANN* method increased the *R*^2^ of the four-individual ocean *LHF* products (ranging from 0.56 to 0.79) to 0.88 and decreased the RMSE (ranging from 19.1 to 37.5) to 11 W m^−2^. Compared to three other *ML* methods (*RF*, *BR* and *RANSAC*), *ANN* method exhibited the best performance according to the validation results. The results of relative uncertainty analysis using the triangle cornered hat *(TCH)* method show that the ensemble *LHF* product using *ML* methods has lower relative uncertainty than individual *LHF* product in most area. The *ANN* was employed to implement the mapping of annual average ocean *LHF* over low-latitude at a spatial resolution of 0.25° during 2003–2007. The ocean *LHF* fusion products estimated from *ANN* methods were 10–30 W m^−2^ lower than those of the four original ocean products (*MERRA*-2, *JOFURO*-3, *ERA*-*I* and *GSSTF*-3) and were more similar to observations.

## 1. Introduction

Ocean latent heat flux (*LHF*) plays a key role in the transformation of energy and vapor at the interface of the atmosphere and ocean [1,2,3]. Knowledge of ocean turbulent fluxes is important for understanding the mechanism of global heat and freshwater budget and is helpful in various research, including on atmospheric issues, oceanic problems and weather prediction. The study of sea–air heat flux can deepen the understanding of the ocean circulation driving model, elucidate the role of the ocean in balancing global energy and develop numerical prediction work on climate change. Both the atmospheric model and the ocean model require accurate *LHF* estimates for numerical simulation and forecasting [4,5,6,7]. Thus, accurate *LHF* estimation of low-latitude regions is essential for climate and hydrology applications. Among them, ocean *LHF* in low-latitude regions has an important impact on global climate change.

Low-latitude areas within 30° N to 30° S, including tropical and subtropical regions, account for approximately half of the Earth’s surface area. Because it is covered by the ocean and receives concentrated solar radiation, low-latitude areas store a large amount of water vapor and heat. The *LHF* transferred from low-latitude oceans to the atmosphere is the main source of atmospheric circulation energy [8,9,10]. Thus, accurate *LHF* estimation of low-latitude areas plays a key role in climate and hydrology applications [11]. Accurate estimation of high spatial resolution ocean *LHF* is vital for researching climate change, and it remains a challenge.

Continuous ocean *LHF* monitoring is mainly located in low-latitude areas, which can improve the accuracy of ocean *LHF* estimates. Many experiments have been carried out to promote the study of air–sea turbulent fluxes [12,13,14,15], such as the Global Energy and Water Cycle Experiment (*GEWEX*) [16] and Joint Global Ocean Flux Study (*JGOFS*) [17]. Based on a large amount of experimental observations, the parameterization scheme of the turbulence flux algorithm has developed rapidly, and global ocean turbulence flux products with different scales and spatial and temporal resolutions have been produced. Satellite and reanalysis data can provide us with spatially and temporally continuous ocean *LHF* observations at various scales. At present, various satellite and reanalysis *LHF* products with moderate or coarse spatial resolution have been produced, including Japanese Ocean Flux Data Sets with Use of Remote Sensing (*J-OFURO*) [18], Goddard Satellite-Based Surface Turbulent Fluxes (*GSSTF-3*) [19], the Hamburg Ocean Atmosphere Parameters and Fluxes from Satellite Data (*HOAPS*) [20], Modern-Era Retrospective analysis for Research and Applications *(MERRA)*, ERA_Interim, etc. However, compared with the observations obtained from buoy sites or experimental ships, satellite-based *LHF* products present large discrepancies. Brunke et al. [21] concluded that satellite-based products have generated large uncertainty in the process of inversion by comparing various turbulent flux products and four satellite products (*GSSTF2*, *GSSSF2b*, *J-OFURO* and *HOAPS*). Some satellite-based products provide accurate *LHF* estimates, but the study areas are limited to specific areas (e.g., *SCS*). Reanalysis products provide us with reasonable estimations of ocean *LHF* and have been successfully used in numerical weather prediction (*NWP*); however, they have notable errors owing to data assimilation schemes. Some reanalysis has a relatively high spatial and temporal resolution but tend to overestimate ocean *LHF* in most areas compared to buoy site measurements. Many studies have also indicated that ocean *LHF* estimates with a coarse spatial resolution (e.g., 1°) may lead to large errors due to the spatial heterogeneity of ocean *LHF* [22].

Over the last forty years, many methods have been developed to implement ocean *LHF* estimates. To date, the methods used for estimating ocean *LHF* can be calculated by: (1) physically-based methods [23,24,25]; (2) data assimilation methods [26,27,28,29,30]; and (3) bulk aerodynamic algorithms [31,32,33,34]. Physically-based methods, including eddy covariance methods and inertial dissipation methods [35,36,37,38], are considered the most reliable methods in estimating ocean *LHF*. However, these methods require high-frequency instruments that can only be implemented for site-scale observations; further, such observations are limited in temporal and spatial distribution. Data assimilation methods can provide a reasonable simulation of the ocean *LHF*, but the difference in the parameterization scheme may introduce significant uncertainty to ocean *LHF* estimations [39,40,41]. Widely used bulk aerodynamic algorithms utilize air temperature, sea surface temperature, air specific humidity and wind speed as input bulk quantities to calculate ocean turbulent fluxes. Even though these methods can be used everywhere on Earth, there are still significant uncertainties from different models [23,42,43]. These methods are widely used to estimate ocean *LHF* at various temporal and spatial resolutions. However, the ocean *LHF* estimates derived from these methods differ substantially from observations [44,45,46].

Recently, multiple product ensembles using machine learning (*ML*) methods have been successfully applied to estimate terrestrial latent heat flux (*LE*). For example, Yao et al. [47] used support vector machine (*SVM*) to integrate three satellite-based *LE* products to improve global terrestrial evapotranspiration (*ET*) estimation and found that the *SVM* method was superior to all other physical methods. Fan et al. [48] developed four tree-based ensemble models (*RF*, *M5Tree*, *GBDT* and *XGBoost*) to estimate daily *ET* using limited meteorological data; the developed XGBoost and GBDT models have accurate predictions, strong model stability and low calculation cost. Shang et al. [49] applied four *ML* methods (Extremely Randomized Trees (*ETR*), Gradient Boosting Regression Tree (*GBRT*), Random Forest (*RF*) and Gaussian Process Regression (*GPR*)) to improve terrestrial *LE* estimations over Europe based on five individual terrestrial *LE* product; the validation results illustrate that the *LE* estimation using *ETR* method increased *R*^2^ and decreased *RMSE*. Even though the *ML* methods have been widely used to estimate terrestrial biophysical variables, there is a lack of experiments on dataset fusion to improve ocean *LHF* estimates by combining multiple *LHF* products.

In this study, we used the Artificial Neutral Network (*ANN*) method to improve ocean *LHF* estimation over low-latitude areas by using four individual *LHF* products. We had three objectives: (1) evaluate the performance of the *ANN* and three other *ML* methods (*RF*, Bayesian Ridge Regression (*BR*) and Random Sample Consensus (*RANSAC*)) by using four *LHF* products based on the moored buoy array of *TAO*, the Research Moored Array for African-Asian-Australian Monsoon Analysis and Prediction (*RAMA*) and the Prediction and Research Moored Array in the Tropical Atlantic (*PIRATA*); (2) assess the relative uncertainties among of the ocean *LHF* products based on the triangle cornered hat (*TCH*) method; and (3) use *ANN* to map the average ocean *LHF* with 0.25° spatial resolution for the period of 2003–2007 by using an ensemble of four *LHF* products.

## 2. Data

### 2.1. Satellite and Reanalysis Ocean LHF Products

The *LHF* products used in this study include the following: *MERRA-2* [50], European Centre for Medium-Range Weather Forecasts (*ECMWF*) interim reanalysis (*ERA-I*) [51], Japanese Ocean Flux Data Sets with Use of Remote Sensing (*J-OFURO*) [18] and Goddard Satellite-Based Surface Turbulent Fluxes (*GSSTF*-3) [19].

Monthly ocean latent heat flux estimates derived from *MERRA-2* [52], with a spatial resolution of 0.5° latitude × 0.625° longitude, from January 2003 to December 2007, was launched aboard the EARTHDATA at https://earthdata.nasa.gov/. The *ERA-I* data [53] were produced by the data assimilation system using 4-dimensional variational assimilation (4D-Var), with a spatial resolution of 0.25° latitude × 0.25° longitude. *J-OFURO* was produced by the School of Marine Science and Technology at Tokai University and was calculated by the COARE 3.0 method with an improved spatial resolution of 0.25°. Compared to the previous versions, *GSSTF*-3 is an improved version with corrected surface specific humidity (Qair) data retrieved by removing the effect of the Earth incidence angle (*EIA*) drifting [23]. These data were obtained from Goddard Earth Science Data and Information Services Center’s (GES DISC) website, with an advantage of a high spatial resolution of 0.25°.

The objectively analyzed air–sea fluxes product (*OAFlux*) [6,54] was used to validate the accuracy of the fusion product. It has been reported that the *OAFlux* dataset is a reliable product for ocean turbulent flux research. The *OAFlux* data have an advantage in that they combine satellite-derived data and reanalysis data by using the objective analysis method; however, these data have a coarser spatial resolution of 1°. To estimate the monthly global ocean *LHF* products at a spatial resolution of 0.25° from 2003 to 2007, we used the bilinear interpolation method. Detailed information for each product mentioned above is summarized in Table 1 and detailed input variable datasets of each *LHF* product is summarized in Table 2.

### 2.2. Buoy Observations

Buoy observations were used as the reference data to evaluate the performance of the ocean *LHF* estimation. The monthly ocean latent heat observations were collected from 115 moored buoy sites [55,56,57,58].

**Figure 1:**
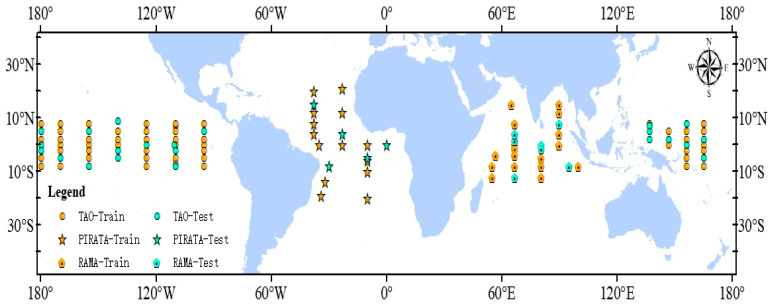
Distribution of 115 buoys sites for different buoy sites arrays over low-latitude area. “Train” represents training sites and “Test” represents the validation sites.

Among 115 moored buoy sites, 67 buoys were collected from the Tropical Atmosphere Ocean/Triangle Trans Ocean Buoy Network (*TAO/TRITON*, https://tao.ndbc.noaa.gov/), 18 buoys were collected from the Prediction and Research Moored Array in the Tropical Atlantic (*PIRATA*, http://www.brest.ird.fr/pirata/) and 12 buoys were collected from the African-Asian-Australian Monsoon Analysis and Prediction (*RAMA*, https://www.pmel.noaa.gov/tao/drupal/disdel/). All ocean turbulent fluxes were calculated by the COARE 3.0 method; these data covered the period from 2003 to 2007. Buoy observations were mainly located in tropic areas. Figure 1 shows buoy site locations and information about the three buoy site arrays. Although the moored buoy footprints varied from the pixel size of the reanalysis and satellite-based products, we still regarded the buoy site observations as “ground truth” in this study.

## 3. Methods

### 3.1. Artificial Neural Network

**Figure 2:**
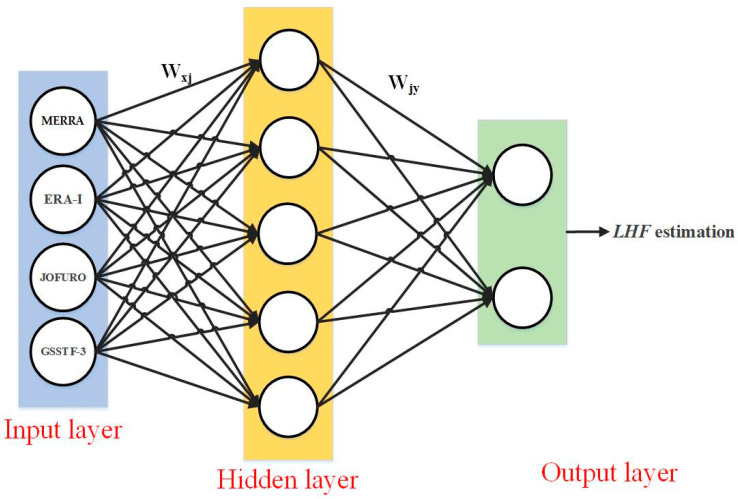
The structure of Artificial neural network (*ANN*) algorithm.

An Artificial Neutral Network (*ANN*) [59] can be considered a network that consists of a series of adaptive connected simple neuron nodes to simulate the human biological nervous system in response to input signals (Figure 2). Different from logistical regression, which is composed of an input and an output layer, the *ANN* comprises three layers: the input layer, the hidden layer and the output layer. Various datasets are considered the input data; the input data are weighted in the hidden layer by means of flexible mathematical algorithms, and the prediction dataset is produced in the output layer. Based on self-adaptation and self-learning, the *ANN* method causes the input and output data to establish a nonlinear relationship.

Within the hidden layer, the fully connected neurons receive input signals from other neurons; these input signals are passed through a weighted connection.

(1) $$y_{j}=\overset{m}{\underset{n\;=\;1}{\sum }}w_{nj}b_{n}$$

where *y_j_* refers to the *j*th neuron output data, *w_nj_* is the connection weight of the *n*th neuron in the hidden layer and the *j*th output layer neuron and *b_n_* is the output of the nth neuron in the hidden layer.

The total input signal value received by the different neurons is used to compare with the threshold of the neuron node,

(2) $$y_{j}=f\left(\beta_{j}-\theta_{j}\right)$$

where *f* is the activation function of the neural network, *β_j_* is the output received by the *j*th neuron in the output layer and *θ_j_* is the threshold of the *j*th neuron in the output layer.

Then, the output data of the neuron are easily generated by the activation function. The learning process of ANN is used to adjust the connection weight between neurons and the threshold of each neuron based on the results of the training data.

### 3.2. Other Machine Learning Methods

#### 3.2.1. Random Forest

*RF* [59] is an ensemble method that is widely applied for regression issues. It uses classification regression tree (*CART*) as a regressor for the decision tree and bootstrap sample methods to select different training datasets for different decision trees. Further, it randomly selects features to perform attribute splitting on internal nodes when constructing a single tree. Therefore, the RF method can better exclude noise interference and has better performance for classification and regression. Generalization is the ability of the Random Forest to correctly predict data outside the training set, and the generalization error is the probability of a misclassification of the data outside the training set by the regression. The generalization error of a Random Forest depends on the regression ability of a single tree and the correlation between any two trees. Research results show that the generalization error of the RF converges to a finite value; thus, as the number of classification trees in the forest increases, the Random Forest does not cause overfitting.

#### 3.2.2. Bayesian Ridge Regression

In addition to the *RF* and *ANN* methods, we also applied two other linear regression methods to predict ocean *LHF* flux. Compared to other *ML* methods, the linear regression method is fast in modeling, and the calculation method is simple. Therefore, even if the amount of input data is large, the calculation speed is fast.

Considering the high correlation among the input variables in this paper, we used the Bayesian Ridge Regression (*BR*) [60] to estimate ocean *LHF* flux. Generally, the linear regression algorithm uses the least squares method to optimize the coefficients. Ridge regression obtains the optimal parameters by penalizing the coefficients to reduce the impact of highly correlated input variables. The Bayesian Ridge Regression (BR) method, which combines the Bayesian method and the ridge regression method, has a strong self-adaptation ability to the input datasets, which not only avoids the overfitting of datasets but also promotes a high utilization rate of data samples.

#### 3.2.3. Random Sample Consensus

The *RANSAC* regression [61] model can obtain valid sample data from the observation dataset containing “outliers” and iteratively estimate the mathematical model parameters. In short, the *RANSAC* method process is as follows: first, randomly extracted samples from the datasets build “interior points”; second, the remaining datasets test the model training by “interior points” and add the sample points that fall within a predetermined tolerance range to the “interior points”; finally, it fits the model with all the “interior points” and uses “interior points” to estimate the error. The process is terminated if the model performance reaches expectations.

### 3.3. Triangle Cornered Hat Method

*TCH* [62] can estimate relative uncertainty without prior knowledge. The *TCH* method is an improved version of the Triangle Cornered (*TC*) method and can be used to calculate the relative uncertainty among three or more independent products. Quantifying uncertainty by removing “true values” from different variables (assuming the input variables all contain true values) is a difference method. This method has been successfully used in gravity fields [63], evapotranspiration [64] and soil moisture [65] at different scales. Here, we applied the *TCH* method to quantify the relative uncertainty among ocean heat fluxes from different products.

The *TCH* method treats the time series of input products as {*X_i_*}, *i* = 1,2, …, *N*. The subscript *i* represents the *i*th product among all sorted products and *N* represents the total number of products. {*X_i_*} can be divided into two parts, including the “true value” {*X_t_*} and the error term {*ε_i_*}:

(3) $$X_{i}=X_{t}+\varepsilon_{i},\;\forall_{i}=1,2,\ldots ,N$$

Due to an unknown true value, it is difficult to obtain the error term {*ε_i_*}. Thus, the first step in the *TCH* method is to determine the difference among the *N* products and reference dataset. First, choose one time series *LHF* product as reference data {*X_R_*} and calculate the differences {*D_i,M_*} between reference data {*X_R_*} and other *LHF* datasets {*X_i_*}.

(4) $$D_{i,M}=X_{i}-X_{R}=\varepsilon_{i}-\varepsilon_{R}\;\forall_{ii}=1,2,\ldots ,N-1$$

where *D_i,M_* represent the difference matrix between the reference dataset {*X_R_*} and the input dataset {*X_i_*}. Next, calculate the (*N* − 1) × (*N* − 1) covariance matrix S = cov (*D*) and determine the covariance matrix of the noise matrix *G* through the S matrix:

(5) $$S=M^{T}\cdot G\cdot M$$

(6) $$M=\left[\begin{array}{c} A\\ -u^{T} \end{array}\right]$$

where *A* is the identity matrix and *u* is the vector of [1 1 1 … 1]. To minimize the global correlation of errors, Premoli and Tavella [66] proposed a free parameter selection criterion to maintain the positive definiteness of *G*. According to the constraint minimization problem proposed by the Kuhn–Tucker theorem, it can be used to determine the unique solution of the matrix *G*, and the random error of each group of data can be calculated.

### 3.4. Evaluation Metrics

The squared correlation coefficient (*R*^2^), root-mean-square-error (*RMSE*) and bias are used as metrics to evaluate the performance of the *LHF* estimations against reference dataset. The matching degree between the evaluated estimations {*x_i_*} and the reference dataset {*r_i_*} can be judged by the metrics mentioned above, and they are written as:

(7) $$R^{2}=\;\frac{{\left(\sum_{i=1}^{N}\left(x_{i}-\bar{x}\right)\left(r_{i}-\bar{r}\right)\right)}^{2}}{\sum_{i\;=\;1}^{N}{\left(x_{i}-\bar{x}\right)}^{2}\sum_{i=\;1}^{N}{\left(r_{i}-\bar{r}\right)}^{2}}$$

(8) $$\mathrm{Bias}=\;\frac{\sum_{i\;=\;1}^{N}\left(x_{i}-r_{i}\right)}{N}$$

(9) $$RMSE=\;\sqrt{\frac{1}{n}\overset{N}{\underset{i\;=\;1}{\sum }}{\left(x_{i}-r_{i}\right)}^{2}}$$

where *N* represents the number of samples. The King–Gupta efficiency (*KGE*) is a comprehensive evaluation metric that can be calculated as follows:

(10) $$KGE\;=\;1-\sqrt{{\left(R-1\right)}^{2}+{\left(\frac{{ST}_{e}}{{ST}_{o}}-1\right)}^{2}+{\left(\frac{E_{e}}{E_{o}}-1\right)}^{2}}$$

where *R* denotes the correlation coefficient between the *LHF* estimation and reference dataset; *ST_e_* and *ST_o_* represent the standard deviation of the *LHF* estimation and reference dataset, respectively; and *E_e_* and *E_o_* are the mean value of the *LHF* estimation and reference dataset, respectively. The closer *KGE* is to 1, the closer the *LHF* estimation is to the reference dataset.

### 3.5. Experimental Setup

Before model construction, we extracted *LHF* variable from four products and in situ measurements. More than 4000 observations from 115 buoy sites were collected as target variable, and *LHF* variable extracted from four products (*JOFURO*-3, *GSSTF*-3, *ERA-I* and *MERRA*-2) were used as predictor variables. To build the model, the datasets (both target dataset and predictor dataset) were randomly divided into two groups: 70% to train the model and the remaining 30% to validate the trained model. The best parameters which can provide the highest correlation coefficient were selected in the training data through cross validation. The obtained optimal parameters were then used in the model to estimate *LHF*.

We constructed the *ANN*, *RF*, *BR* and *RANSAC* model based on sklearn modules by using the Python platform. The main parameters of *ANN* models include the learning_rate, hidden_layer_sizes, n_estimators and min_samples_split. The performance of *RF* method in the scikit-learn toolbox is mainly influenced by n_estimators and max-features. The main parameters of *BRR* are n_iter and lambda. The main parameters to adjust when using *RANSAC* are max_trials, min_samples and residual_threshold. Obtaining the optimal parameters of the model can not only improve the accuracy of model estimation but also improve efficiency and shorten model running time.

To find the optimal parameter for each *ML* method, we applied the GridSearchCV module. GridSearchCV method is a parameter tuning method. It tries every possibility through loop traversal among all parameter combinations and selects the optimal parameter combination based on the performance of the results. The main disadvantage of this method is that it is time-consuming. Optimal parameter combinations for each *ML* methods were determined by GridSearchCV method in optional parameters, as shown in Table 3.

## 4. Results

### 4.1. Validation of the Five Ocean LHF Products against Buoy Observations

**Figure 3:**
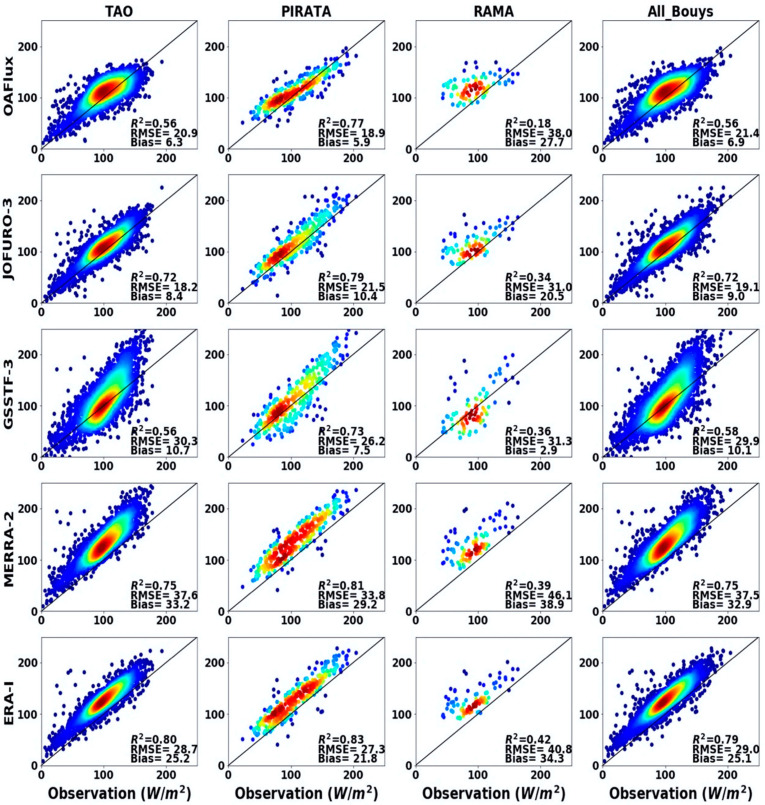
Validation of five products against buoy observations from 115 sites during the period from 2003 to 2007 (unit W m^−2^).

At the site scale, the five ocean *LHF* products exhibited substantial differences in ocean *LHF* estimation, as shown in Figure 3. For the *TAO* buoy site array with the most observations, the monthly ocean *LHF* estimation of *ERA-I* product correlated best with the observations, indicated by an *R*^2^ = 0.80 (*p* < 0.01); however, the *RMSE* and bias both exceeded 25.2 W m^−2^. Similarly, *MERRA* also showed good performance with an *R*^2^ > 0.75 (*p* < 0.01) but highly overestimated ocean *LHF* as indicated by the highest *RMSE* and bias among all ocean *LHF* products. In contrast, the *OAFlux* product showed relatively lower *RMSE* and the lowest bias; the lower correlation (*R*^2^ = 0.56, *p* < 0.01) may be caused by its coarse spatial resolution (approximately 1°). *GSSTF-3* performed least satisfactorily in estimating ocean *LHF*, with disperse distribution of validation points and relatively high overall estimates. Compared to other observation arrays, all ocean *LHF* products performed better with higher *R*^2^ and lower bias in the *PIRATA* buoy site array.

**Figure 4:**
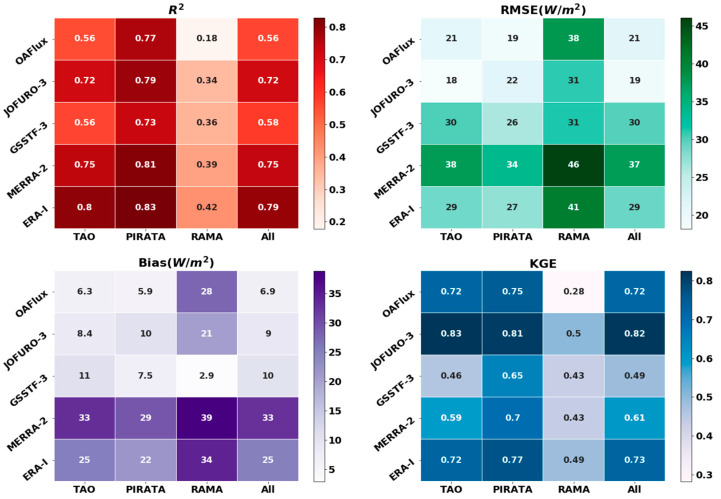
The evaluation parameters (*R*^2^, *RMSE*, bias and *KGE*) comparison between the five ocean *LHF* products against buoy sites observations (unit W m^−2^).

Figure 4 shows the *R*^2^, *RMSE*, bias and *KGE* statistics of five ocean *LHF* products against observations from different buoy sites. For all buoy site arrays, reanalysis products (*MERRA* and *ERA-I*) have the highest *R*^2^ ranging from 0.39 to 0.83. However, the magnitude of average monthly *LHF* derived from reanalysis is much higher than that of buoy-measured *LHF*, as indicated by biases exceeding 25 and 32 W m^−2^, respectively. When considering the *KGE* (ranging from 0.5 to 0.83) and *RMSE* (ranging from 18 to 31 W m^−2^), the *JOFURO-3* product is superior to others. This indicates that different parameterizations of ocean *LHF* products affect the accuracy of ocean *LHF* estimates. The performance of *OAFlux* products is lower than *JOFURO-3* but better than *GSSTF-3*; this is probably caused by the coarse spatial resolution of *OAFlux*, which has a spatial resolution of 1°.

### 4.2. Ensemble of Four Ocean LHF Products from ANN and Other ML Methods

#### 4.2.1. Model Training and Validation based on Buoy Observations

None of the individual ocean *LHF* products provides the best *LHF* estimates based on buoy observations. Thus, we used *ANN* and other *ML* methods (*RF*, *BR* and *RANSAC*) to calculate ocean *LHF* by an ensemble of four ocean *LHF* products: *MERRA*, *ERA-I*, *JOFURO*-3 and *GSSTF*-3. The reanalysis products are highly correlated with measurements but also highly overestimate ocean *LHF*. In contrast, the satellite-based product *JOFURO*-3 and objectively analyzed product *OAFlux* perform well with lower bias.

**Figure 5:**
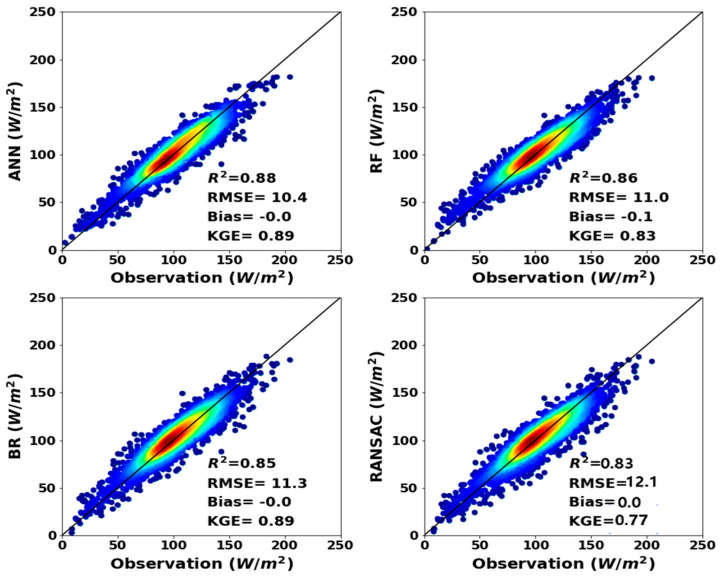
The scatter plots for ocean *LHF* observations at 81 training buoy sites and *LHF* estimates from the *ANN*, *RF*, *BR* and *RANSAC* methods during 2003–2007 (unit W m^−2^).

Figure 5 represents the training results of *ANN*, *RF*, *BR* and *RANSAC* for all buoy site observations. Estimated ocean *LHF* derived from four *ML* methods agreed well with buoy measurements and is consistent with the trend of buoy observations. Among the four *ML* methods, *ANN* has the highest *R*^2^ and lowest *RMSE* in training datasets, as indicated by an *R*^2^ exceeding 0.88. The *RANSAC* method has a slightly higher *RMSE* of 12.1 W m^−^^2^ and a slightly lower *R*^2^ than other *ML* methods.

**Figure 6:**
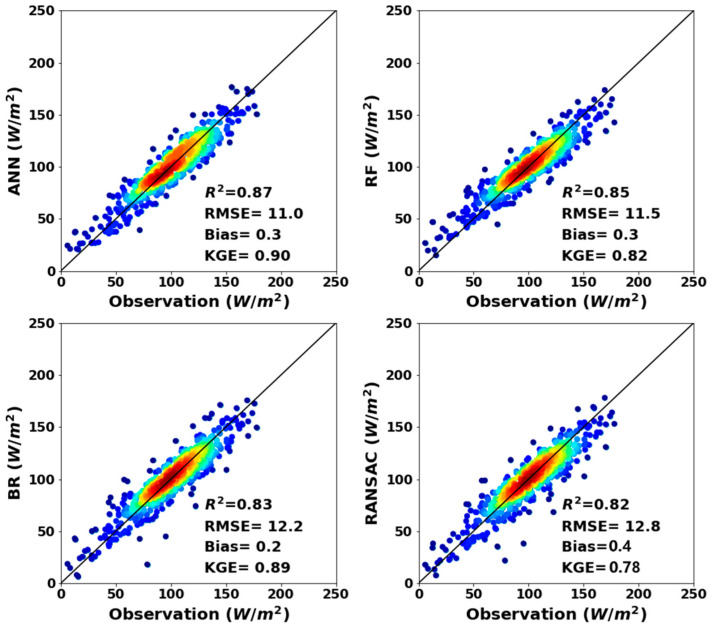
The scatter plot for ocean *LHF* observations from 34 validation buoy sites and the *LHF* estimations from the *ANN*, *RF*, *BR* and *RANSAC* methods during 2003–2007 (unit W m^−2^).

Figure 6 shows the scatter plot for the ocean *LHF* observations and *LHF* estimations from four *ML* methods. The validation results show that *ANN* yields the best estimations of ocean *LHF*, as indicated by the highest *R*^2^ of 0.87, the lowest *RMSE* of 10.9 W m^−2^ and the highest comprehensive index (*KGE*) of 0.90, followed by *RF* and *BR*. Although *RANSAC* performed weakly as indicated by the lowest *R*^2^ (0.82) and *KGE* (0.78), it is still superior to any individual ocean *LHF* product. Overall, these results illustrate that the ocean *LHF* fusion products derived from *ML* methods are superior to individual *LHF* products. In addition, the *ANN* model performs best among the four *ML* models.

#### 4.2.2. Relative Uncertainties of Ocean *LHF* Over Low-latitude Areas

To quantify the performance of all four methods over the tropics, we used the *TCH* method to calculate the uncertainties of ocean *LHF* estimates derived from *ML* methods.

**Figure 7:**
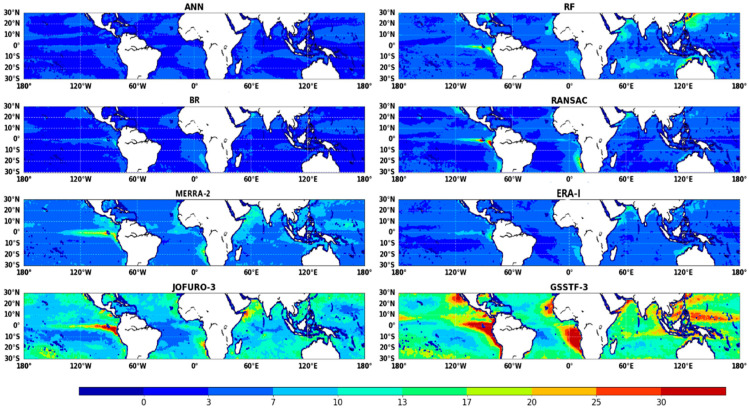
Distribution of relative uncertainties from eight ocean *LHF* products (including four ocean *LHF* products and four ensemble *LHF* products) over low-latitude area (unit W m^−2^).

Figure 7 presents the distribution of relative uncertainties estimated from ocean *LHF* estimations based on four *ML* models. Generally, the ocean *LHF* estimated from *ML* methods perform better than those from ocean *LHF* products (*MERRA*, *ERA*-*I*, *JOFURO*-*3* and *GSSTF*-*3*). Moreover, the *LHF* products tend to generate lower uncertainties in the area away from the coast due to the stable and uniform climatic conditions and have higher uncertainties in the area close to land. The *ANN* and *BR* perform well over low-latitude areas with lower relative uncertainties; *BR* has higher uncertainties than *ANN* in the west of South Africa and equatorial area. Among the *LHF* estimations based on *ML* methods, *RF* has the highest relative uncertainties, especially in the Kuroshio current region and Southern Hemisphere Subtropical area, which may be caused by the errors in the model estimation. For ocean *LHF* products, although the *ERA-I* outperforms the other three *LHF* products, it underperforms the *ANN* estimation in most areas.

**Figure 8:**
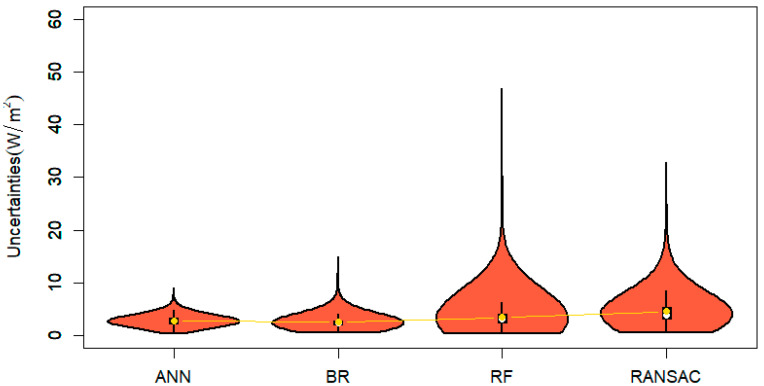
Violin plots of relative uncertainties from four ensemble ocean *LHF* products over low-latitude area (unit W m^−2^).

Figure 8 shows the relative uncertainties of ocean *LHF* estimations calculated by four *ML* methods. *ANN* outperforms the other *LHF* estimations with lower average relative uncertainties of 2.60 W m^−2^, a median value of 2.39 W m^−2^ and a maximum of relative uncertainties lower than 10 W m^−2^. The *BR* is second to *ANN* with higher average relative uncertainties of 2.86 W m^−^^2^ and a maximum of relative uncertainties of 20 W m^−2^. Although the median values of relative uncertainties for *RF* and *RANSAC* are slightly higher than *ANN* as indicated by values of 3.15 and 3.95, respectively, they have higher relative uncertainties exceeding 30 W m^−2^.

Overall, *ANN* slightly outperformed other *ML* methods according to validation against buoy observations and relative uncertainty evaluation based on the *TCH* method over tropical areas.

### 4.3. Mapping of Ocean LHF Over Low-Latitude Areas

#### 4.3.1. Annual Patterns of the Ensemble Tropical Ocean LHF

**Figure 9:**
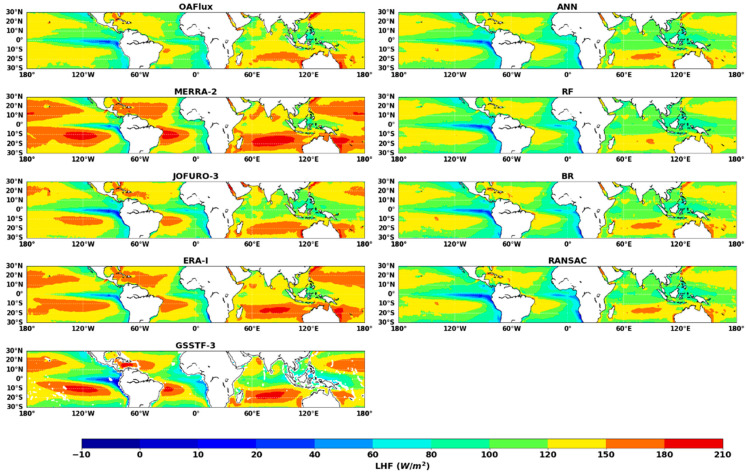
Maps of multi-year (2003-2007) annual average ocean *LHF*. The first column (left) presents the maps of ocean *LHF* from *OAFlux*, *MERRA-2*, *JOFURO-3*, *ERA-I* and *GSSTF-3*. The second column presents the maps of ocean *LHF* using *ANN*, *RF*, BR and *RANSAC* (unit W m^−2^).

We applied *ANN*, *RF*, *BR* and *RANSAC* driven by monthly ocean *LHF* products (*MERRA-2*, *JOFURO-3, ERA-I* and *GSSTF-3*) to estimate ocean *LHF* in low-latitude areas at 0.25° spatial resolution from 2003 to 2007. Figure 9 shows the spatial distribution of annual *LHF* averaged from different products during the years 2003–2007.

All of the ocean *LHF* products yielded lower *LHF* estimates over the equatorial region, especially over the East Pacific along South America and the East Atlantic along Africa. The highest ocean *LHF* exceeded approximately 200 W m^−2^ and occurred in the South Pacific with latitudes between 10° and 20° S. Even though ocean *LHF* products have similar spatial distribution in low-latitude areas, there are still significant differences between different products. As shown in Figure 9, *MERRA-2* and *GSSTF-3* products yielded higher *LHF* values in the South Pacific and South Indian Ocean. Compared with *MERRA-2*, *JOFURO-3, ERA-I* and *GSSTF-3*, *OAFlux* yielded lower ocean *LHF* values over low-latitude areas. In contrast, the spatial distribution of *ANN*, *BR* and *RANSAC* showed highly consistent characteristics. However, it is also noted that the ocean *LHF* estimation from *RF* cannot properly simulate the high ocean *LHF* values, which may be because the *RF* algorithm highly depends on the representativeness of the sample dataset; if the sample dataset does not include the high ocean *LHF* value, it may cause a deviation in *LHF* results. The ocean *LHF* of four ensemble products is much lower than that from the four individual ocean products, but close to that from the reference dataset (*OAFlux* product).

**Figure 10:**
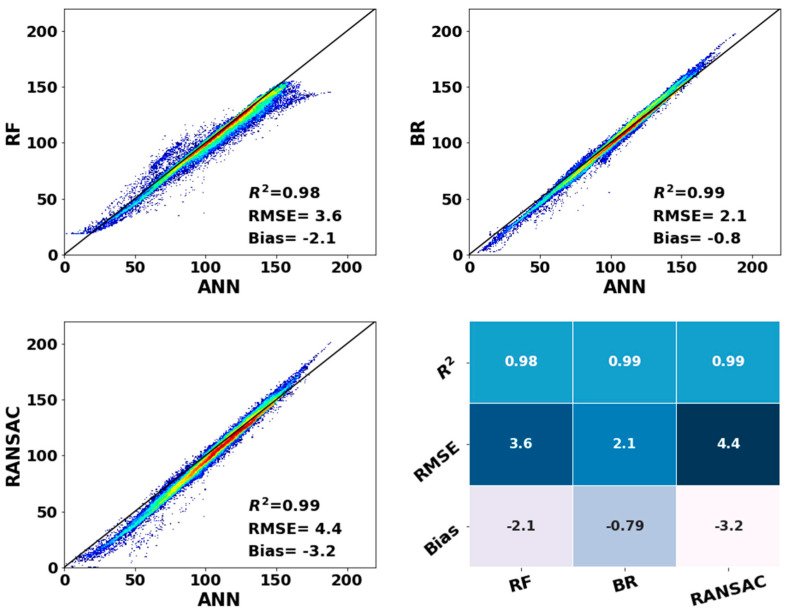
Comparison of monthly ocean *LHF* from ANN and other *ML* methods (unit W m^−2^).

Figure 10 shows the comparison of the annual average *LHF* from *ANN* versus that of the other three methods (*RF*, *BR* and *RANSAC*) over low-latitude areas. In general, the ocean *LHF* from *ANN* agrees best with *BR*, followed by *RANSAC*. The most prominent difference between *ANN* and *BR* is that the *LHF* values estimated by *ANN* were lower than those of *BR* when *LHF* was greater than 150 W m^−2^ and higher than those of *BR* when it was less than 50 W m^−2^. When the ocean *LHF* was lower than 10 W m^−2^, *RANSAC* poorly simulated *LHF* variability. The estimated *LHF* from *ANN* was higher than those of *RF* when ocean *LHF* was greater than 130 W m^−2^ and lower than those of *RF* when it was less than 20 W m^−2^. The discrepancies may be mainly caused by the difference in algorithm structure. As shown in Figure 9, the estimated ocean *LHF* from *ANN* was close to that from the other three *ML* methods, indicated by an *R*^2^ higher than 0.98 and bias less than 3.5 W m^−2^. As mentioned above, the estimated *LHF* from *BR* was closest to that of *ANN*, as characterized by the highest *R*^2^, the lowest *RMSE* and lowest bias.

**Figure 11:**
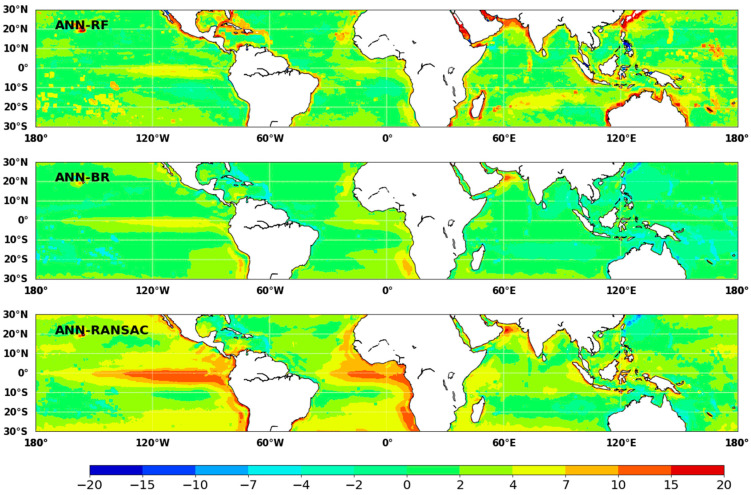
Maps of spatial difference in annual average ocean *LHF* between *ANN* and other *ML* methods (*RF*, *BR* and *RANSAC*) over low-latitude area during 2003–2007 (unit W m^−2^).

Figure 11 shows spatial differences in annual ocean *LHF* over low-latitude regions between *ANN* and the other three *ML* methods (*RF*, *BR* and *RANSAC*). The differences in annual ocean *LHF* estimated by *ANN* and the other three *ML* methods were mainly distributed in the range of −10 to 10 W m^−2^. The ensemble ocean *LHF* product from *BR* also showed a consistent spatial distribution with that of *ANN*; the difference was less than 5 W m^−2^ in most areas. The ensemble *LHF* using *RANSAC* has the most significant variation in spatial distribution. *RANSAC* yields high ocean *LHF* mainly in the Equatorial East Pacific and Equatorial East Atlantic; this may be caused by the differences in different *ML* methods.

#### 4.3.2. Seasonal Patterns of the Ensemble Tropical Ocean LHF

**Figure 12:**
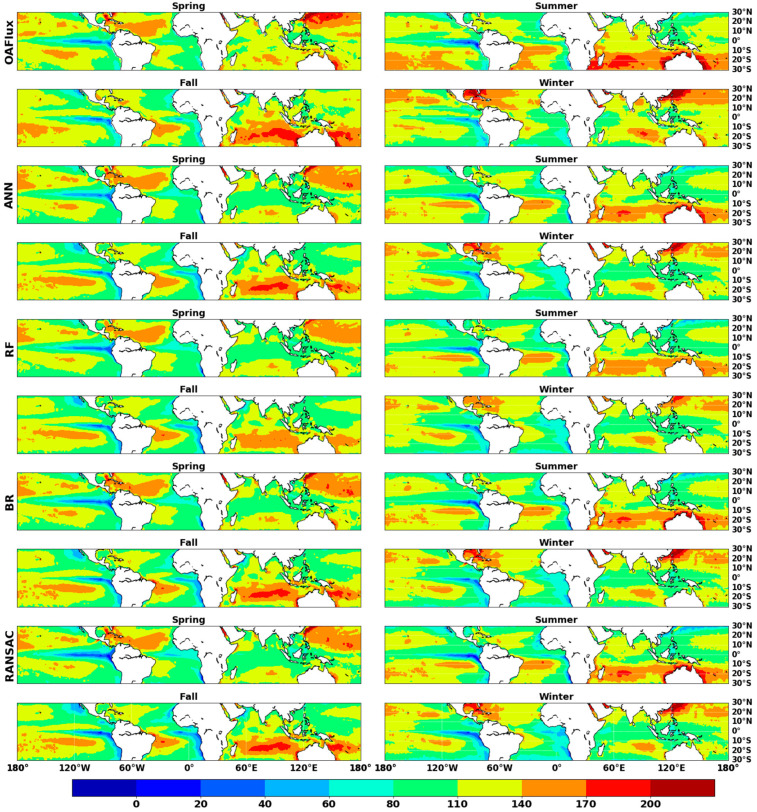
Maps of multiyear (2003–2007) average seasonality of ocean *LHF* estimations from *OAFlux* product and four *ML* methods (unit W m^−2^).

Figure 12 presents the multiyear average seasonal pattern of ocean *LHF*, which shows that strong regional variations occur in low-latitude areas. The variation in ocean *LHF* is affected by the climate and land–sea distribution. Lower wind speeds and lower sea temperatures due to proximity to land result in the lowest ocean *LHF* in the equatorial current region of the eastern Pacific. Moreover, the maximum ocean *LHF* occurs in the central Pacific.

In the northern hemisphere, ocean *LHF* increased from fall then decreased from spring to fall over tropical areas. The lowest ocean *LHF* in the southern hemisphere occurs in spring; there was a sharp increase in ocean *LHF* from spring to fall. In the winter of the northern hemisphere, the largest ocean *LHF* occurs in the Kuroshio Current, followed by the Gulf Stream; the average ocean *LHF* exceeds 200 W m^−2^ in these regions. Ocean *LHF* values are large in the Kuroshio Current and Gulf Stream due to the large temperature differences at the air–sea interface. Similarly, the largest ocean *LHF* of the southern hemisphere occurs in the South Pacific, especially in the 10° and 20° south latitude.

Compared to the *RF* method, the *ANN* method exhibited a good ability to simulate spatial variability. *RF* performed poorly in simulating high ocean *LHF* in ocean current regions, such as the Kuroshio Current area in the northern hemisphere’s winter, and Australia’s bordering sea. This may be caused by the fact that *RF* has a different algorithm structure compared to those of the other *ML* methods. According to the *ANN* method, fall has the highest average ocean *LHF* (113.8 W m^−2^), followed by summer (113.0 W m^−2^), spring (112.7 W m^−2^) and winter (111.2 W m^−2^).

**Figure 13:**
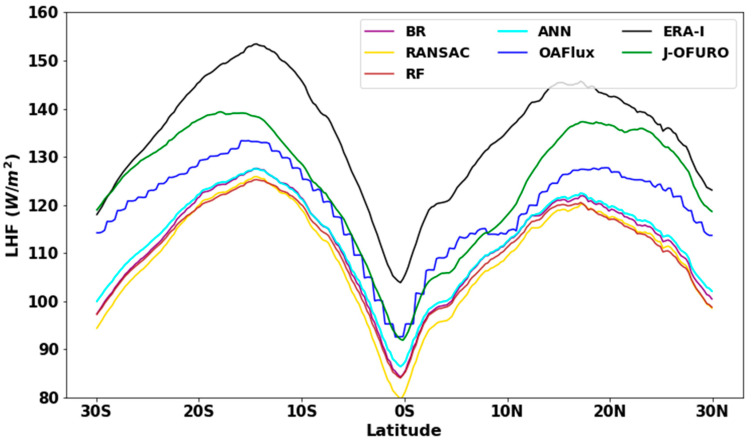
Latitudinal variation of annual average *LHF* over low-latitude area during 2003–2007, (unit W m^−2^).

Figure 13 illustrates the latitudinal variation in annual average *LHF* over low-latitude areas during 2003–2007. Despite the general differences in latitude distribution among different ocean *LHF* estimates, the latitudinal distribution of all ocean *LHF* estimations is bimodal, and the highest ocean *LHF* occurs at approximately 15° S, followed by 15° N. The minimum values appear in the equatorial region, and the *LHF* gradually increases with the increase in latitude. After reaching the maximum value at approximately 15°, ocean *LHF* gradually declines to the poles. There are still substantial differences between the seven ocean *LHF* estimates. Compared to reanalysis datasets that overestimate ocean *LHF* against buoy site observation, *ANN* is closer to *OAFlux* and *JOFURO*-*3*. Moreover, *ANN*-based ensemble *LHF* can capture more detailed information than OAFlux owing to its high spatial resolution. Although the estimated ocean *LHF* of four *ML* methods are very close, the estimated *LHF* using *ANN* is slightly higher than others by 3–5 W m^−2^.

**Figure 14:**
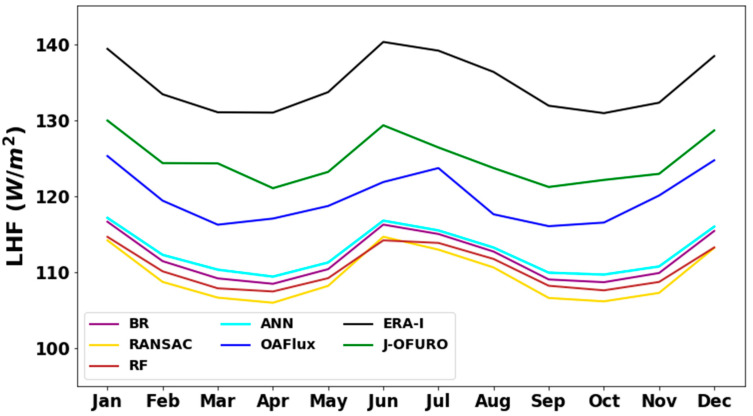
Annual variation of annual average *LHF* over low-latitude regions during the period of 2003 to 2007 (unit W m^−2^).

Figure 14 compares the monthly average ocean *LHF* derived from *ANN* and the other *ML* methods with other ocean *LHF* products over the tropics. All the ocean *LHF* estimates presented similar seasonal variability, and the magnitude of ocean *LHF* seasonal variation was less than 10 W m^−2^. Ocean *LHF* increased from April to June then decreased from June to October due to the high ocean *LHF* in the southern hemisphere. Figure 13 also illustrates that the reanalysis product (*ERA-I*) was 10–30 W m^−2^ higher than others. The *LHF* derived from the four *ML* methods was closer and more than 10 W m^−2^ lower than those derived from the other products. Among them, *ANN* is slightly higher than other *ML* methods by 1–3 W m^−2^.

## 5. Discussion

*ANN*, *RF*, *BR* and *RANSAC* were used to estimate ocean *LHF* at a spatial resolution of 0.25°. Some of these four *ML* methods were successfully used to estimate a terrestrial LE fusion product, such as *ANN* and *RF* [49,67,68]. According to validation against buoy observations (Figure 4), our results illustrate that ensemble ocean *LHF* from *ML* methods performed much better than the estimated *LHF* from four individual ocean *LHF* products (*MERRA-2*, *JOFURO-3*, *ERA-I* and *GSSTF*-3). Compared to the individual *LHF* products, the *R*^2^ of the *ML* methods was 3.7–46.4% higher and the bias decreased by approximately 15 W m^−2^. Our results also show that some minor differences existed among the four *ML* methods, which are mainly affected by the structure of different fusion algorithms [69,70]. Sagi and Rokach [71] showed that the differences in structure of the ensemble methods may significantly affect the predictions, and the best ensemble method for a given problem needs to consider other factors (such as suitability to a given setting).

The ensemble ocean *LHF* from *ANN* showed great consistency with that of the other three *ML* methods. The difference in the spatiotemporal variation of ensemble *LHF* from the four *ML* methods was less than 5 W m^−2^ and were all 10–30 W m^−2^ lower than the individual ocean *LHF* products. In comparison with the *RF*, *BR* and *RANSAC* methods, the ocean *LHF* estimation using *ANN* performed better. This may be attributed to the fact that *ANN* is composed of a series of adaptively connected simple neuron nodes, which improves the accuracy of model estimation by adjusting the weights between different neurons [59,72]. Other studies also found that *ANN* presents a superior ensemble performance to other *ML* methods in many fields, such as *ET*, solar radiation and downscaling [67,73,74].

Similar to *ANN*, *RF* has a strong correlation with observations (Figure 4), but we found that ocean *LHF* estimations from *RF* performed poorly in simulating high *LHF* values in the spatiotemporal distribution. This is consistent with the conclusions drawn by Zhang et al. [75]; the regression prediction obtained by *RF* performs poorly, while *ANN* achieves the best estimation of the total biomass in four *ML* methods. This may be due to the fact that the training of *RF* not only requires a large amount of sample dataset [76] but also requires the sample dataset itself to be representative. Studies have shown that increasing the number and periodicity of sample datasets can improve the estimation accuracy of *RF* [77]. Considering that the buoy site observations cannot cover all the ocean *LHF* features in the study area, the *RF* method underperformed the other three *ML* methods in simulating the spatial and temporal distribution of *LHF*.

We also applied the *TCH* method to evaluate relative uncertainties among the four ocean *LHF* fusion products and four individual *LHF* products because the *TCH* method has been successfully used in territorial *LE* uncertainty evaluation [78,79,80]. All *ML* methods performed better than individual ocean *LHF* products as indicated by lower relative uncertainty. The relative uncertainty of the four ensemble products was approximately 5 W m^−2^, while relative uncertainty of the individual ocean *LHF* products ranged from 7 to 20 W m^−2^. *ANN* had the lowest average uncertainty, followed by *BR*. The average relative uncertainty of *RF* was the highest among the four *ML* methods.

In terms of the spatial distribution of relative uncertainty, the high relative uncertainty values of *RF* were mainly located in the extreme values of ocean *LHF* (Figure 7 and Figure 12). The uncertainties of the ensemble *LHF* products mainly stemmed from the biases of the individual datasets [21], the errors in the buoy site observations [81], the mismatched spatial scales between datasets from different sources and the structure of *ML* methods [71,82,83]. Due to the measurement sensors and environmental disturbances, the uncertainties in ocean *LHF* observations obtained from buoy sites array were approximately 10 W m^−2^ [44,56,81]. The representativeness of the buoy site ranges from tens to hundreds of meters, while the spatial resolution of *LHF* products was greater than 12.5 km, the spatial resolution mismatch may lead to uncertainties in the validation results. Additionally, the errors in the individual products will lead to an 8% error in ensemble *LHF* [21,84]. The mismatches among different data sources may also introduce a 5–7% uncertainty in ensemble *LHF* [85,86,87]. Although the *ML* methods do not require a priori knowledge, the structure of different *ML* methods may lead to large errors and poor generalization performance [84].

Our study provided future efforts to improve ocean *LHF* estimation using *ML* methods by an ensemble of multiple *LHF* products. *ML* methods performed better in estimating ocean *LHF* than the individual ocean *LHF* products (*MERRA-2*, *JOFURO-3*, *ERA-I* and *GSSTF-3*). All these products can be well trained by observations and then used for estimating ocean *LHF*. Importantly, different *ML* methods need to be fully evaluated in different studies. For example, *RF* has higher relative uncertainty at the region of extreme *LHF* values, but that is not conclusive. In contrast, *ANN* presents lower relative uncertainty in global or regional ocean *LHF* estimations. Therefore, *ANN* can be considered an ideal method to replace *RF* when generating tropical ocean *LHF* products.

## 6. Conclusions

We applied *ANN* and three other *ML* methods (*RF*, *BR* and *RANSAC*) to improve tropical ocean *LHF* estimation by ensemble of satellite and reanalysis products (*MERRA-2*, *JOFURO-3*, *ERA-I* and *GSSTF-3*) and evaluate the performance of fusion products based on reference product (*OAFlux*) and buoy observations. The *ML* models used here were trained (tested) using observations from 81 (34) buoy sites over low-latitude areas from 2003 to 2007.

By merging individual *LHF* product, our results show that the ensemble *LHF* products derived from four *ML* methods were significantly superior to the individual *LHF* products with higher accuracy and lower bias. Among them, *ANN* performs best, indicated by the highest *R*^2^ (0.88 and 0.87), the lowest *RMSE* (10.4 and 10.9) and the highest *KGE* (0.89 and 0.90) for training and testing, respectively.

By quantifying relative uncertainties by the TCH method, we found that the relative uncertainties of ensemble LHF products were also significantly lower than individual LHF product, which lead to the conclusion that the individual product’s uncertainties caused by errors in algorithm and input datasets can be reduced by merging multiple products. In addition, *ANN* generated lower relative uncertainty than the other three *ML* methods. The result demonstrates that *ANN* can be considered an ideal method to replace *RF* when generating tropical ocean *LHF* products.

## Figures and Tables

**Table 1:** Summary of the five ocean *LHF* products in this study for 2003–2007.

Products	Variables	Spatial Resolution	Time Span	References
MERRA-2	*LHF*	1/2° × 2/3°	1980–present	Rienecker et al., 2011
ERA-interim	*LHF*	0.125°	1979–present	Dee et al., 2011
GSSTF-3	*LHF*	0.25°	1987–2008	Shie 2012
J-OFURO	*LHF*	0.25°	1988–2013	Tomita et al., 2018
OAFLUX	*LHF*	1°	1958–present	Yu et al., 2004

**Table 2:** Summary of input variable datasets for each *LHF* product.

Product	Variable	Source
**MERRA-2**	Surface winds	SSM/I; QuikSCAT; ERS (ERS-1 and ERS-2);
	Rain rate	SSM/I; TRMM Microwave Imager (TMI);
	Radiances	SSM/I; GOES sounder; TIROS Operational Vertical Sounder (TOVS) and Advanced TOVS (ATOVS); AIRS; MSU; AMSU-A;
	Upper-level winds	geostationary satellites and MODIS
	ozone	SBUV
**ERA-I**	Surface winds; Ocean wave height	ERS (ERS-1 and ERS-2);
	Radiances	VTPR; High Resolution Infrared Sounder (HIRS); Stratospheric Sounding Unit (SSU); MSU; AMSU-A;
	Upper-level winds	Meteosat-2
	Ozone profiles	SBUV
	clear-sky radiances	Meteosat-2
	Surface wind speed; Column water vapor	SSM/I
	radio occultation (RO)	CHAMP; COSMIC; GRACE
**GSSTF-3**	wind speed (U)	SSM/I
	surface air specific humidity (Qair)	corrected SSM/I brightness temperature (Tb)
	Radiances	SIRS, HIRS, VTPR, and TOVS;
	Upper-level winds	geostationary satellites
**JOFURO-3**	wind speed (U)	SSM/I; TMI; WindSAT; AMSR-E; AMSR2; ERS (ERS-1 and ERS-2); QuikSCAT; ASCAT-A; ASCAT-B; OSCAT
	Qair	SSM/I; TMI; AMSR-E; AMSR2
	SST	MGDSST; OSTIA-NRT; AMSR-E; MW; OISST; AMSR; TMI; WindSAT; GMI; OSTIA-RA
**OAFlux**	wind speed (U)	SSM/I; AMSR-E; QuikSCAT;
	Qair	SSM/I;
	SST	NCEP-OI; NCEP–NCAR; NCEP–DOE; ERA-40

**Table 3:** Parameters setting to determine the optima parameters for *ML* methods.

Method	Parameters	Optional	Interval	Selection of GridSearchCV
***ANN***	activation	“identity”, “logistic”, “tanh”, “relu”	-	relu
	learning_rate	“10”, “1”, “0.1”, “0.01”, “0.001”, “0.0001”	-	0.01
	hidden_layer_sizes	“(10, 50)”, “(10, 100)”, “(20, 80)”, “(20,150)”, “(30,100)“, “(50,100)”	-	(20, 80)
	batch_size	50–300	50	150
***RF***	min_samples_split	2–6	1	4
	n_estimators	5–50	3	40
	max-features	1–10	1	6
***BR***	n_iter	20–300	20	240
	lambda	“0.1”, “0.01”, “0.001”, “0.0001” “0.00001”, “0.000001”, “0.0000001”	-	“0.000001”
***RANSAC***	max_trials	30–120	5	105
	min_samples	80–300	20	260
	residual_threshold	5–100	5	45
